# A genome-wide association study identified candidate regions and genes for commercial traits in a Landrace population

**DOI:** 10.3389/fgene.2024.1505197

**Published:** 2025-01-06

**Authors:** Guojian Ma, Xihong Tan, Ying Yan, Tianyang Zhang, Jianhua Wang, Xiaoling Chen, Jingya Xu

**Affiliations:** ^1^ Breeding Department, Wuhan COFCO Meat Co., Ltd., Wuhan, Hubei, China; ^2^ COFCO Nutrition and Health Research Institute, Beijing, China

**Keywords:** pigs, feed efficiency, backfat thickness, GWAS, candidate genes

## Abstract

Backfat thickness (BFT) and feed conversion ratio (FCR) are important commercial traits in the pig industry. With the increasing demand for human health and meat production, identifying functional genomic regions and genes associated with these commercial traits is critical for enhancing production efficiency. In this research, we conducted a genome-wide association study (GWAS) on a Landrace population comprising 4,295 individuals with chip data for BFT and FCR. Our analysis revealed a total of 118 genome-wide significant signals located on chromosomes SSC1, SSC2, SSC7, SSC12, and SSC13, respectively. Furthermore, we identified 10 potential regions associated with the two traits and annotated the genes within these regions. In addition, enrichment analysis was also performed. Notably, candidate genes such as *SHANK2*, *KCNQ1*, and *ABL1* were found to be associated with BFT, whereas *NAP1L4*, *LSP1*, and *PPFIA1* genes were related to the FCR. Our findings provide valuable insights into the genetic architecture of these two traits and offer guidance for future pig breeding efforts.

## Introduction

The increasing demand for human health and food nutrition has become a challenge due to the rapid growth of global population. Consequently, enhancing the production efficiency of livestock products has become vital for the livestock industry and sustainable development (Mehrabi et al., 2020). Swine is one of the most important economic livestock in the world, providing a diverse range of products to meet human needs. The rapid development of breeding methods, such as genomic selection, has effectively reduced the genetic interval between pig generations and significantly improved the performance of commercial pig breeds by increasing prediction accuracy (Knol et al., 2016). As consumers’ demand for healthier meat products increases, pigs have been bred for lower fat content and higher lean meat. Previous research uncovered that daily energy intake is related to whole-body fat composition in male pigs (Liu et al., 2021), and leaner pigs tend to exhibit higher feed efficiency. Therefore, understanding the genetic architecture of these commercial traits is essential.

Feed conversion ratio (FCR) and backfat thickness (BFT) are primary commercial phenotypes in the pig industry and have been extensively analyzed by numerous researchers. Candidate genes, such as phospholipase A2 group IB (*PLA2G1B*), have been reported to be associated with feed efficiency by influencing lipid catabolism (Fu L. et al., 2020; Hollie and Hui, 2011). Additionally, the members of the insulin-like growth factor family, such as *IGF1* and *IGF2*, have been found to affect the growth rate and feed conversion efficiency (Zhu et al., 2014). Backfat thickness is another important trait in pig production as it impacts lean meat yield and the popularity of pork meat (Hoa et al., 2021). Many loci on SSC1, SSC5, SSC6, SSC7, and SSC12, as well as candidate genes such as *MC4R*, *IGF2*, and *LEPR*, were found to be related to backfat thickness (Gozalo-Marcilla et al., 2021; Fu M. et al., 2023).

Over the past 15 years, genome-wide association studies (GWASs) have been employed to investigate the linkage between genomic markers and records of various traits (Abdellaoui et al., 2023). This approach has facilitated the identification of numerous quantitative trait nucleotides (QTNs) and candidate genes associated with FCR and BFT (Li W. et al., 2022; Delpuech et al., 2021; Ding et al., 2022; Miao et al., 2023), which provided deep insights into these commercial traits and improved the quality of meat production. To date, about 55,688 quantitative trait loci (QTLs) have been released by pig QTLdb (Hu et al., 2005). However, due to the complexity of these quantitative traits, many QTLs remain unknown.

Using a Landrace population with genomic chip data, a total of 4,295 individuals with two important commercial traits, including BFT at 100 kg and FCR, were analyzed in this study. Related variants with annotated candidate genes within candidate regions were detected using a mixed-effects linear model in a genome-wide association study. Furthermore, linkage disequilibrium (LD) block analysis with candidate regions and enrichment analysis of candidate genes were also performed. The main objectives of this research were to identify the associated genomic regions and candidate genes of BFT and FCR within our population. In addition, we also used a multi-omics swine database (Fu Y. et al., 2020) to prioritize the candidate genes in order to provide an understanding of the majority of candidate genes.

## Results

### Summary of phenotype and genotype data

**FIGURE 1:**
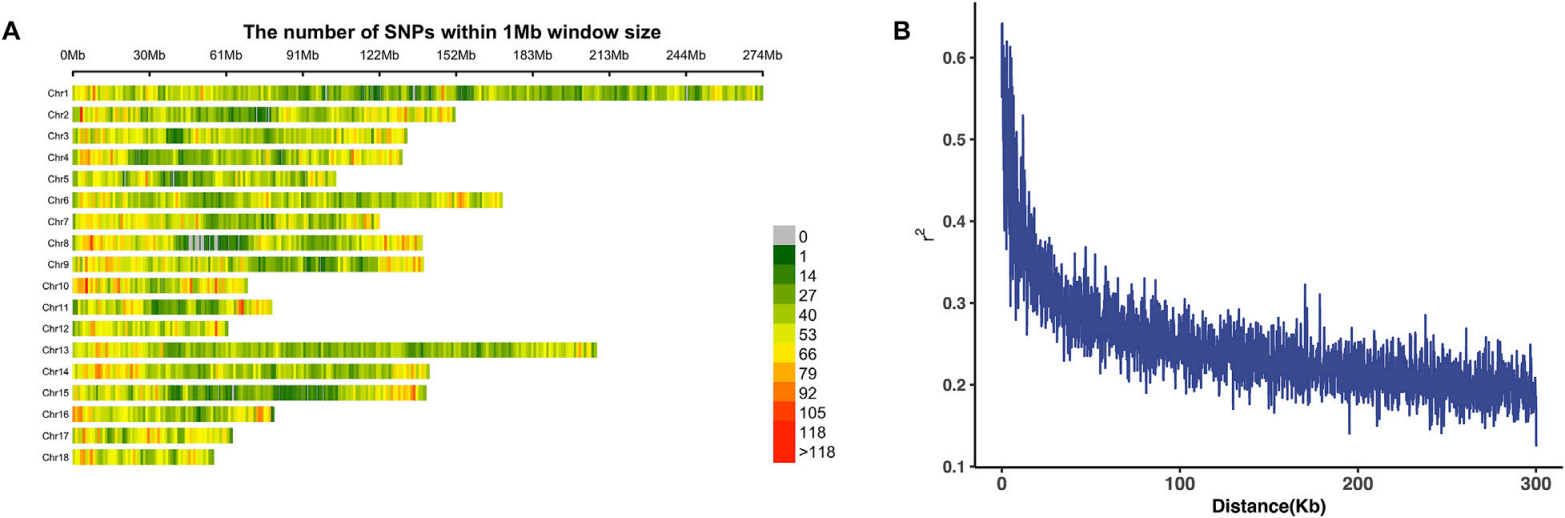
Density plot of genome variants and LD decay plot. **(A)** Genotype density plot of whole genome variants. The SNPs were counted using a 1-Mb window size, and the legend shows different colors representing the number of SNPs. **(B)** LD decay plot of the Landrace population.

In this research, we used a Landrace population consisting of 4,295 individuals with chip-level genotype data derived from a functional SNP lipid chip. Summary statistics, including the sample size, mean of phenotype values, standard deviation of phenotypes, and coefficient of variation, are provided in Table 1. Additionally, phenotype distribution plots are shown in Supplementary Figure 1. The mean values for BFT (100 kg) and FCR in our population are 10.22 and 2.3 with standard deviations of 1.99 and 0.22 and the coefficients of variation are 0.2 and 0.09, respectively. According to Table 1 and Supplementary Figure 1, both traits can be used for further analysis. Quality control was performed on the genomic data. After imputation and filtering, 100,235 SNPs on autosomes remained for the association study, excluding those with a minor allele frequency (MAF) less than 0.01. The marker density on each chromosome is shown in Figure 1A. LD decay analysis was also conducted using PopLDdecay (Zhang et al., 2019), and the results are shown in Figure 1B.

**TABLE 1:** Summary statistics of phenotypes.

Phenotype	Number	Mean	SD^a^	CV^b^
BFT100 kg	4,295	10.22	1.99	0.2
FCR	4,295	2.3	0.22	0.09

^a^ SD, standard deviation of each phenotype.

^b^ CV, coefficient of variation for each phenotype.

### Genome-wide association studies

**FIGURE 2:**
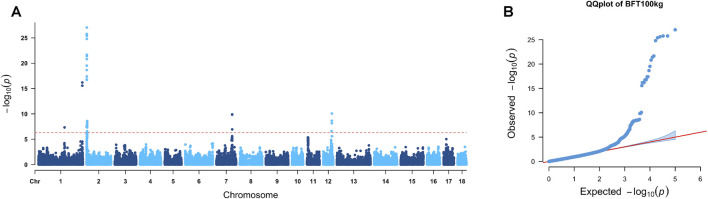
GWAS results of BFT (100 kg). **(A)** In the Manhattan plot of backfat thickness, the red line represents the Bonferroni cutoff, which was 0.05/N, and N represents the number of variants used in the analysis. **(B)** QQ plot for the GWAS of backfat thickness. The *x*-axis represents the expected $-\log_{10}\left(P\right)$ , and the *y*-axis represents the observed $-\log_{10}\left(P\right)$ .

Genome-wide association studies were performed through a mixed-effects linear model in the rMVP package (Yin et al., 2021). Sex, farms, and the first three principle components were included as fixed effects, whereas the additive genetic effects were considered the random effects variable. A Bonferroni cutoff of 0.05/*N* was used as a significant threshold, where *N* represents the number of SNPs. The details of quality control and genotype data are described in Methods. A total of 69 significant SNPs distributed on SSC1, SSC2, SSC7, and SSC12 were identified to be associated with backfat thickness. The details of these SNPs are shown in Supplementary Table 1. Manhattan and quantile–quantile (QQ) plots for BFT (100 kg) are shown in Figure 2, with a lambda value of 0.93 indicating minimal population inflation in GWAS. Based on the LD decay results, a distance of 300 kb was determined to define the candidate regions around each significant signal, and the candidate regions are shown in Table 2. Regions with overlapping areas were merged into one region, and a total of 244 genes were annotated within these candidate regions. After annotation, the genes were prioritized by a multi-omics database called ISwine (Fu Y. et al., 2020). Further details are provided in Supplementary Table 3.

**TABLE 2:** Summary of the identified candidate regions and genes of phenotypes.

Phenotype	Chr	Candidate region	Candidate gene
BFT100 kg	1	160,473,437–161,073,437	*MC4R*
	1	270,269,333–270,926,968	*ABL1* and *FIBCD1*
	2	0–2,457,364	*ENSSSCG00000035293*, *SLC22A18*, *PTDSS2*, *HRAS*, *PNPLA2*, *TALDO1*, *BRSK2*, and *DUSP8*
	2	2,758,419–3,644,831	*ANO1*, *FGF19*, *FGF3*, *FGF4*, *SHANK2*, *KCNQ1*, and *INS*
	7	97,275,068–98,039,684	*AREL1* and *VRTN*
	12	50,709,260–53,168,040	*ARRB2*, *GLTPD2*, *TNFSF12*, *SLC16A13*, and *TP53*
FCR	1	262,745,504–263,345,504	*ENSSSCG00000035556*, *ENSSSCG00000031416*
	2	133,461–733,461	*RNH1*, *TSPAN4*, *AP2A2*, and *RASSF7*
	2	945,702–3,644,831	*KCNQ1*, *ANO1*, *SHANK2*, *LSP1*, *PPFIA1*, *NAP1L4*, *IFITM10*, *TNNI2*, *PRR33*, *TNNT3*, *MOB2*, *INS*, *SYT8*, *ASCL2*, *CTSD*, *TH*, and *DUSP8*
	13	44,899,972–45,499,972	*SYNPR*

**FIGURE 3:**
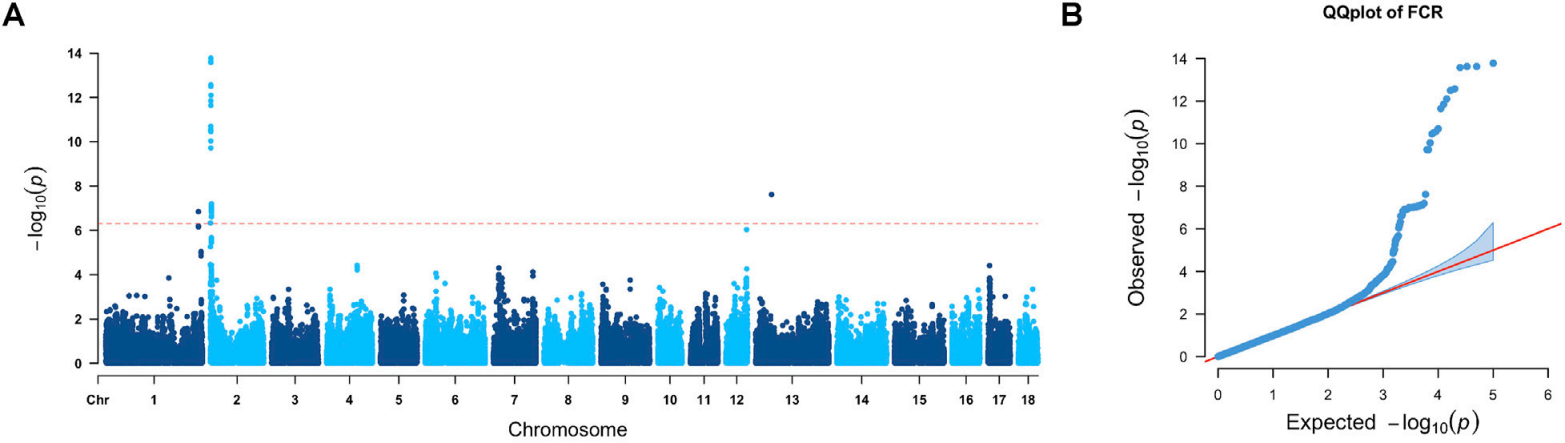
GWAS results of the FCR. **(A)** In the Manhattan plot of the feed conversion ratio, the red line represents the Bonferroni cutoff, which was 0.05/N, and N represents the number of variants used in the analysis. **(B)** QQ plot for the GWAS of the FCR. The *x*-axis represents the expected $-\log_{10}\left(P\right)$ , and the *y*-axis represents the observed $-\log_{10}\left(P\right)$ .

A total of 49 significant markers located on SSC1, SSC2, and SSC13, respectively, were found to be related to the FCR. Manhattan and QQ plots of FCR GWAS results are presented in Figure 3, where the lambda value was 0.96 for FCR GWAS results. The candidate regions and genes for the FCR are provided in Table 2, and the details of gene annotation in the candidate regions are displayed in Supplementary Table 4.

### Gene ontology annotation analysis and enrichment results

The candidate genes were annotated with Gene Ontology (GO) and the Kyoto Encyclopedia for Genes and Genomes (KEGG) database using IAnimal (Fu Y. et al., 2023). The details for the gene annotation results of BFT are shown in Supplementary Figure 2 and Supplementary Table 5. A total of 161 GO terms were significantly enriched, comprising 94 biological processes (BPs), 21 cellular components (CCs), and 46 molecular functions (MFs). Notably, several significant GO terms were associated with backfat thickness, such as positive regulation of insulin secretion (*P* = 0.019), intermembrane lipid transfer (*P* = 0.028), and lipid transfer activity (*P* = 0.041). Significant pathways, such as the MAPK signaling pathway (*P* = $3.49{\times 10}^{-3}$ ), were also found in KEGG results. Genes such as anoctamin 1 (*ANO1*), glycolipid transfer protein domain containing 2 (*GLTPD2*), TNF superfamily member 12 (*TNFSF12*), fibroblast growth factor 19 (*FGF19*), fibroblast growth factor 3 (*FGF3*), fibroblast growth factor 4 (*FGF4*), and *ENSSSCG00000035293* were involved in these significant terms and pathways.

Enrichment results for the FCR are shown in Supplementary Figure 3 and Supplementary Table 6. A total of 37 KEGG pathways were significantly enriched (*P* < 0.05). We also found 132 significant GO terms, including 94 BPs, 12 CCs, and 26 MFs. The positive regulation of insulin secretion, involved in the cellular response to glucose stimuli, is associated with nutrient absorption and energy metabolism, which were significantly enriched (*P* = 0.0057), and anoctamin 1 (*ANO1*) was involved in this process. Furthermore, some digestion-related pathways, such as protein digestion and absorption, were detected with suggestive *P*-values. Potassium voltage-gated channel subfamily Q member 1 (*KCNQ1*) was involved in this pathway and has been reported to be related to pig feed efficiency (Xiang et al., 2024).

### Linkage disequilibrium block analysis

**FIGURE 4:**
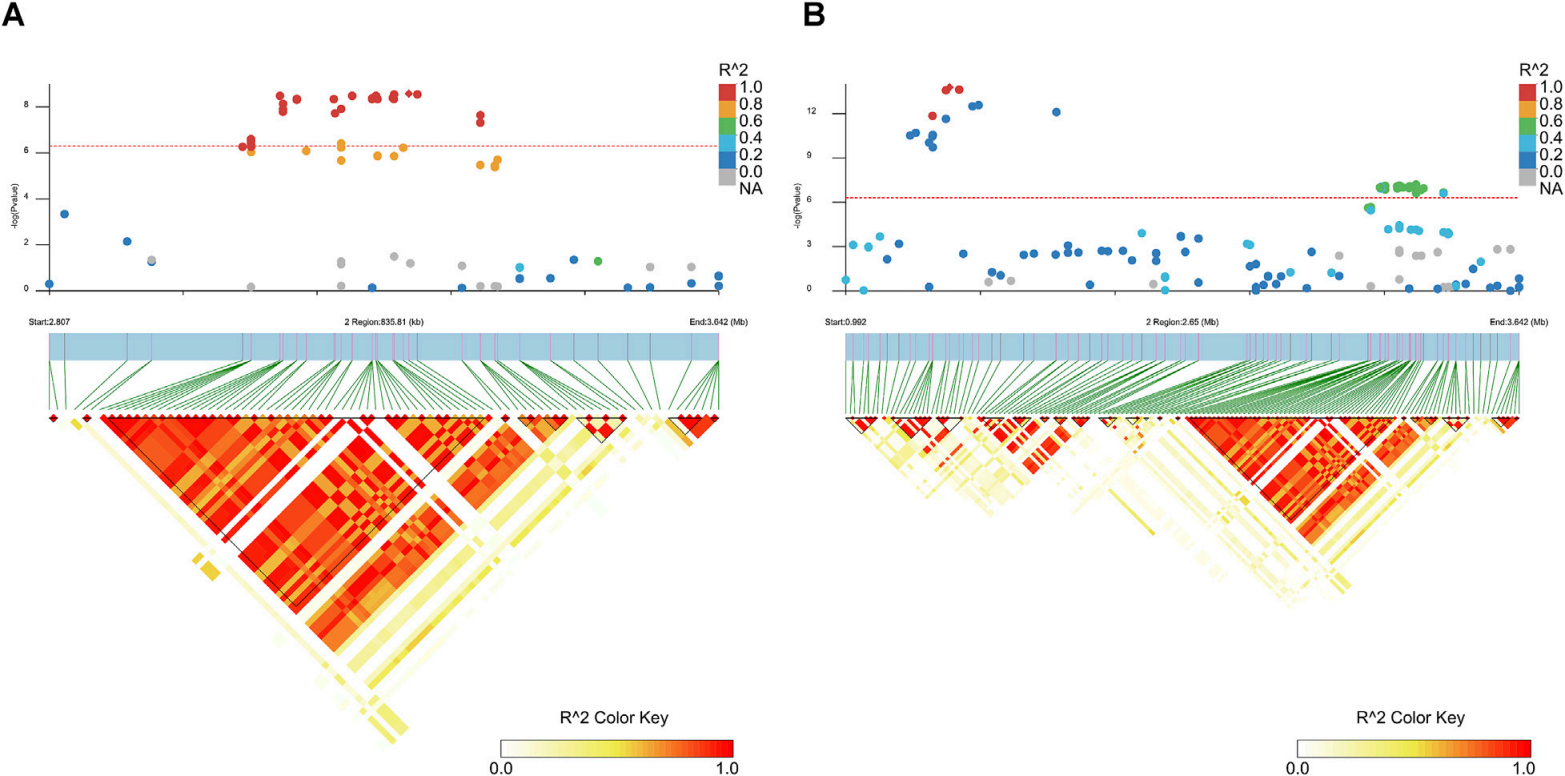
Linkage disequilibrium plot for SNPs within the most significant candidate regions. **(A)** Linkage disequilibrium plot for SNPs within Chr2: 2,758,419–3,644,831, which is the candidate region for BTF (100 kg). The red line represents the Bonferroni cutoff, which was 0.05/*N*, where *N* represents the number of variants used in the analysis. The legend on the right side represents the different R^2^ values of SNPs around the regions of peak SNP detection. **(B)** Linkage disequilibrium plot for SNPs within Chr2: 945,702–3,644,831, which is the candidate region for BTF (100 kg); the red line represents the Bonferroni cutoff, which was 0.05/*N*, where *N* represents the number of variants used in the analysis. The legend on the right side represents the different R^2^ values of SNPs around the regions of peak SNP detection.

The LD blocks around the peak signals were analyzed and plotted using LDBlockShow (Dong et al., 2020). These plots are shown in Figure 4, Supplementary Figure 4, and Supplementary Figure 5. Figure 4 highlights the most significant regions of GWAS results for the two traits. Multiple LD blocks were observed around the top signals, with an overlapping region between BFT and FCR results, spanning from 2.76 to 3.64 Mb on SSC2, indicating that this region may have an influence on both FCR and BFT.

## Discussion

In our research, the GWAS approach was used to identify related genomic regions and candidate genes for backfat thickness and feed conversion ratio; a total of 4,295 Landraces were involved and analyzed. Our results showed 10 candidate regions on the genome for two commercial traits, and among the identified candidate genes, we found many reported genes that should be related to these two traits.

There were 46 overlapping signals between the GWAS results for BFT and FCR, suggesting that these variants may have influences on both traits. Some genes in these candidate regions were found to be related to both backfat thickness and feed conversion ratio. For example, as a member of the SHANK protein family, *SHANK2* (SH3 and multiple ankyrin repeat domains 2) was reported to be associated with childhood obesity (Comuzzie et al., 2012). It was strongly highlighted as a candidate gene for backfat thickness in an association study based on imputed whole-genome data from a multi-breed population (Li J. et al., 2022). Interestingly, *SHANK2* was found to be associated with average daily gain and the meat-to-fat ratio in pooled F2-designed pigs (Falker-Gieske et al., 2019). Since *SHANK2* was also found as a candidate gene for the FCR, it may play an important role in both backfat thickness and feed conversion ratio.

The MAPK signaling pathway was significantly enriched in the KEGG analysis results for backfat thickness (*P* = $3.49{\times 10}^{-3}$ ), which was reported to be essential for adipogenesis as it can regulate porcine fat deposition (Aouadi et al., 2006; Zhang et al., 2021; Wang et al., 2022). A total of nine genes, namely, *FGF3*, *FGF4*, *FGF19*, *ARRB2*, *INS*, *HRAS*, *DUSP8*, *TP53*, and *ENSSSCG00000035293*, were involved in this pathway. Many of these genes have been reported in previous studies. For example, *INS* was identified as a candidate gene for porcine backfat thickness (Gozalo-Marcilla et al., 2021). It has been reported that the blood glucose level is regulated by insulin, which is encoded by the *INS* gene; it can also promote cell fat storage and affect lipid metabolism (Saltiel and Kahn, 2001). Additionally, *DUSP8* was found to be related to ham weight loss, and *HRAS* was reported to be associated with backfat in pigs. Other genes related to fat metabolism, such as *PTDSS2*, *TALDO1*, and *BRSK2*, have been identified in recent studies (Gozalo-Marcilla et al., 2021; Faggion et al., 2024; Blaj et al., 2018). *ABL1* was detected as a candidate gene for backfat traits in Yorkshire and Duroc populations (Ma et al., 2019; Li et al., 2021). It was also found to be related to the meat-to-fat ratio in pigs (Falker-Gieske et al., 2019). A recent GWAS in the Yorkshire population also confirmed that *ABL1* was associated with the average daily gain (Park, 2024). The QTL on SSC1 (270 Mb) was similar to the *FIBCD1* gene, which was also found in a Swiss Large White pig population and associated with the body mass index in humans (Nosková et al., 2022; Pulit et al., 2019). *SLC16A13* and *PNPLA2* were found in the candidate region on SSC2. *SLC16A13* is a candidate gene for diabetes in mice, and its deletion will attenuate lipid accumulation and insulin resistance (Schumann et al., 2021). *SLC22A18* is also related to lipids; knocking down *SLC22A18* in mice will reduce hepatic lipid accumulation, revealing its positive effects on lipid accumulation (Yamamoto et al., 2024). *PNPLA2* promoted lipid accumulation in an adipogenesis test in pigs (Wang et al., 2018).

*MC4R* is a major gene influencing fatness in pigs. It is also involved in the regulation of feeding behavior and body weight in mice and humans. A missense mutation in this gene leads to increased fat accumulation in pigs (Ovilo et al., 2006). *MC4R* has also been shown to affect growth, feed intake, and backfat thickness in pigs, according to previous studies by Gozalo-Marcilla et al. (2021), Lee et al. (2020), Galve et al. (2012), and Piórkowska et al. (2010). We also found that *AREL1* and *VRTN* on SSC7, which have previously been associated with body length, teat number, and intramuscular fat content (Hong et al., 2021; Park et al., 2023; Hirose et al., 2013; Yang et al., 2016), are also associated with meat production traits, thus influencing backfat.

Pigs are known for their outstanding olfactory abilities, which are attributed to their abundant functional olfactory receptors (Groenen et al., 2012). Odors affect pig reproduction and also have an influence on early food preferences (Brunjes et al., 2016). In the region of 262,745,504–263,345,504 on SSC1, we detected *ENSSSCG00000035556* and *ENSSSCG00000031416* to be associated with the FCR. These genes were enriched in an MF term of olfactory receptor activity (*P* = 0.015). In addition to these two genes, nine other genes (*ENSSSCG00000036003*, *ENSSSCG00000032805*, *ENSSSCG00000032825, ENSSSCG00000027589*, *ENSSSCG00000037454, ENSSSCG00000027732*, *ENSSSCG00000031516, ENSSSCG00000026287*, and *ENSSSCG00000035439*) were also enriched in this term and may be related. However, based on the prioritization results, *ENSSSCG00000035556* and *ENSSSCG00000031416* had higher scores, suggesting that these two genes may have a more significant impact on the FCR. The KEGG pathway analysis also identified significant enrichment in an olfactory-related pathway, namely, olfactory transduction (*P* = 0.046). *ANO1* (anoctamin 1) was part of this pathway, along with 11 other genes enriched for olfactory receptor activity, as mentioned earlier. Olfactory receptors can perceive odor, transform biochemical signaling events into electrical impulses, and send them to the brain (Ma, 2007). This pathway was also found to be significantly enriched and related to residual feed intake (Do et al., 2014). Therefore, *ANO1* may affect the FCR through olfactory-related pathways. *ANO1* also affects fat deposition in pigs (Shi et al., 2022).

Feed efficiency can be largely influenced by energy metabolism and digestion. *KCNQ1* (potassium voltage-gated channel subfamily Q member 1) was the most significantly related gene among these candidate genes. It plays an important role in fetal development in mice and humans, and it was reported to be associated with development. *KCNQ1* is an imprinted gene expressed paternally in pigs (Wu et al., 2020) that influences nutrient absorption by regulating gastric acid secretion, as well as salt and glucose homeostasis (Sun and MacKinnon, 2020). It is also found to be involved in a related KEGG pathway that facilitates the digestion and absorption of proteins. In a recent multi-omics study, *KCNQ1*, along with *SYT8*, *TNNI2*, *ASCL2*, *MOB2*, *DUSP8*, *TH*, *PRR33*, *TNNT3*, *IFITM10*, *CTSD*, and *INS*, was identified as a candidate gene for the FCR in Large White boars (Xiang et al., 2024). These genes were also observed in our results. Interestingly, *KCNQ1* was investigated in an association study as one of the candidate genes in the backfat thickness of pigs (Lee et al., 2018). The *LSP1* gene has been reported to be associated with weight loss after dry-curing of hams (Faggion et al., 2024). Additionally, it can stimulate myogenic factors and influence skeletal muscle development in pigs (Albuquerque et al., 2021). Since skeletal muscle plays a key role in energy storage and consumption and is closely linked to energy metabolism, *LSP1* may impact muscle development, thereby affecting pig growth and FCR. *TSPAN4* and *AP2A2* were also reported to be associated with growth in pigs with *LSP1* (Faggion et al., 2024). In addition to *LSP1*, *PPF1A1* was found to be differently expressed in the top three canonical pathways (Liu et al., 2015). In an epigenome-wide skeletal muscle study, *NAP1L4* was detected to have CpG positions hypermethylated within its promoters (Ponsuksili et al., 2019). *RASSF7* is a candidate gene for the FCR and a member of the N-terminal Ras association domain family. Studies have shown that knocking down *RASSF7* restricts cell growth (Recino et al., 2010), and a deficiency in lysine negatively impacts the expression of *RASSF7* (Wang et al., 2017). A mitochondrial protein, RNH1, was found to be associated with angiogenesis in porcine corpus luteum (Likszo et al., 2021). Synaptoporin (*SYNPR*) is one of the tetratransmembrane transport vesicle proteins, which is distributed in the digestive system (Liu et al., 2022). It has been found to be genome-wide associated with autoimmune hepatitis (*AIH*) in humans (Li Y. et al., 2022). We identified *SYNPR* as one of the significant candidate genes on SSC13. Therefore, it may affect feed efficiency through digestion.

In conclusion, we performed a GWAS based on a Landrace population to investigate two commercial traits. Our analysis identified 118 significant signals, from which 10 candidate regions were selected. Candidate genes within these regions were annotated and further analyzed using GO and KEGG pathways. Among the identified candidate genes, *MC4R*, *SLC22A13*, and *INS* were associated with backfat thickness, whereas *ENSSSCG00000035556*, *SHANK2*, *KCNQ1*, and *LSP1* were related to the feed conversion ratio. Overall, our research provides deeper insights into the genetic basis of these traits and could inform future pig breeding efforts.

## Materials and methods

### Collection of Landrace population and phenotypes

A total of 4,295 Landrace individuals were used in this research, collected from three different great-grandparent farms of COFCO Joycome Foods Co., Ltd. All the pigs were raised under uniform feeding and management standards during the measurement period. Original records, including daily feed intake and weight for each pig, were automatically collected using the Pig Performance Testing System (Nedap, Groenlo, Netherlands). Outliers in these records were removed. The start and end dates of the test, along with the initial and final weights, weight gain, and total feed consumed during the test, were recorded. The FCR was then calculated as the total feed intake divided by the weight gain. The backfat thickness was measured using living B-ultrasonography at the end of the test, and the measured traits were then adjusted to a body weight of 100 kg. Details and distribution of phenotypes are shown in Table 1 and Supplementary Figure 1.

### Genotyping, imputation, and quality control

Genomic DNA was extracted from ear tissue samples using the Tecan Freedom EVO NGS Workstation and the MagPure Tissue DNA KF Kit (MD5112-02), with a concentration of ≥40 ng/µL and a quantity of ≥1 µg. After that, the samples were genotyped using the Porcine 80K functional SNP genotyping chip by Wuhan Yingzi Gene Co., Ltd., using target capture sequencing technology. A total of 187,255 variants were included in the original genotype data. Quality control was performed using PLINK 1.90 (Purcell et al., 2007). Markers with a call rate <90% and MAF < 0.01 and variants on the sex chromosomes were excluded. This resulted in the retention of 100,240 variants, with a total genotyping rate of 0.99. Beagle 5.4 (Browning et al., 2018) was applied to impute genotype data. After imputation, quality control was performed again to remove markers with MAF < 0.01. A total of 100,235 SNPs and 4,295 individuals were left for further analysis at last.

### Genome-wide association study

GWAS analysis was performed by fitting a mixed-effects linear model using the following equation in the rMVP (Yin et al., 2021) package:

y=Xβ+Vα+Zu+e,

where y represents the phenotypic values for FCR and BFT (100 kg); β represents the fixed effects, including the first three principal components, sex, and farm; α represents the SNP vector being tested; $u\;∼\;N\left(0,G\sigma_{u}^{2}\right)$ represents the vector of random effects; and $e\;∼\;N\left(0,\sigma_{e}^{2}\right)$ represents the vector of residual errors. In this study, the additive genetic relationship matrix is imputed by the genomic relationship matrix G , derived from all SNP variants used in the association test (VanRaden, 2008), and X , V , and Z are the incidence matrices for β , α , and u , respectively. A significant threshold of 0.05/*N* was confirmed by a Bonferroni correction, where *N* represents the number of SNPs. Manhattan and QQ plots were constructed in the R environment using rMVP.

### Identification of candidate regions, genes, and enrichment analysis

LD decay analysis was performed to detect the size of the candidate region using PopLDdecay (Zhang et al., 2019). A window size of 300 kb was determined based on the LD decay results. Significant SNPs were then annotated to nearby genes within a 300 kb upstream or downstream range and prioritized using the multi-omics swine knowledgebase ISwine (Fu Y. et al., 2020). The candidate regions were uploaded in the section “Search by Region” on ISwine to obtain a candidate gene list. Then, we used “Prioritize” in the tool section to prioritize the candidate genes for each commercial trait and downloaded the results. Enrichment analyses, including GO and KEGG pathway analyses, were performed using enrichment tools from the IAnimal database (Fu Y. et al., 2023). The LD blocks around the top QTN plot were generated by LDBlockShow (Dong et al., 2020).

## Data Availability

The datasets presented in this study can be found in online repositories. The names of the repository/repositories and accession number(s) can be found at: https://figshare.com/, https://figshare.com/articles/dataset/genotype_data_of_Landrace_GWAS/27861177.

## Supplementary material

SUPPLEMENTARY TABLE 1 Details of GWAS results for BFT.

SUPPLEMENTARY TABLE 2 Details of GWAS results for FCR.

SUPPLEMENTARY TABLE 3 Details of annotated genes for BFT.

SUPPLEMENTARY TABLE 4 Details of annotated genes for FCR.

SUPPLEMENTARY TABLE 5 Details of GO and KEGG results for BFT.

SUPPLEMENTARY TABLE 6 Details of GO and KEGG results for FCR.
