# Supplementary material for: A genome-wide association study identified candidate regions and genes for commercial traits in a Landrace population
Source: Front Genet. 2025 Jan 6;15:1505197. doi: 10.3389/fgene.2024.1505197 (PMC11743953; doi:10.3389/fgene.2024.1505197)
Supplement: Supplementary file 3 [file Supplementaryfile1.pdf]

Supplementary materials

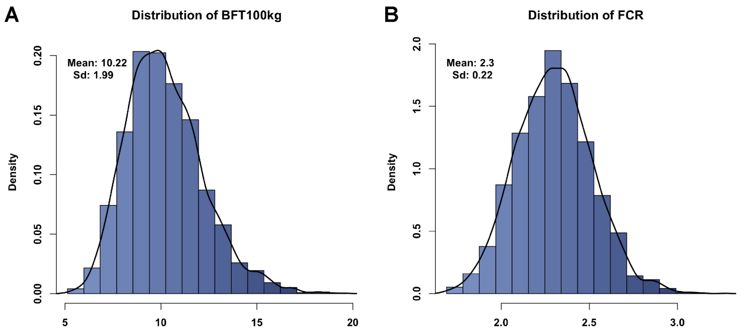

Supplementary Figure 1. Phenotype distribution plots. (A) Phenotype distribution plot for backfat thickness. (B) Phenotype distribution plot for feed conversion ratio.

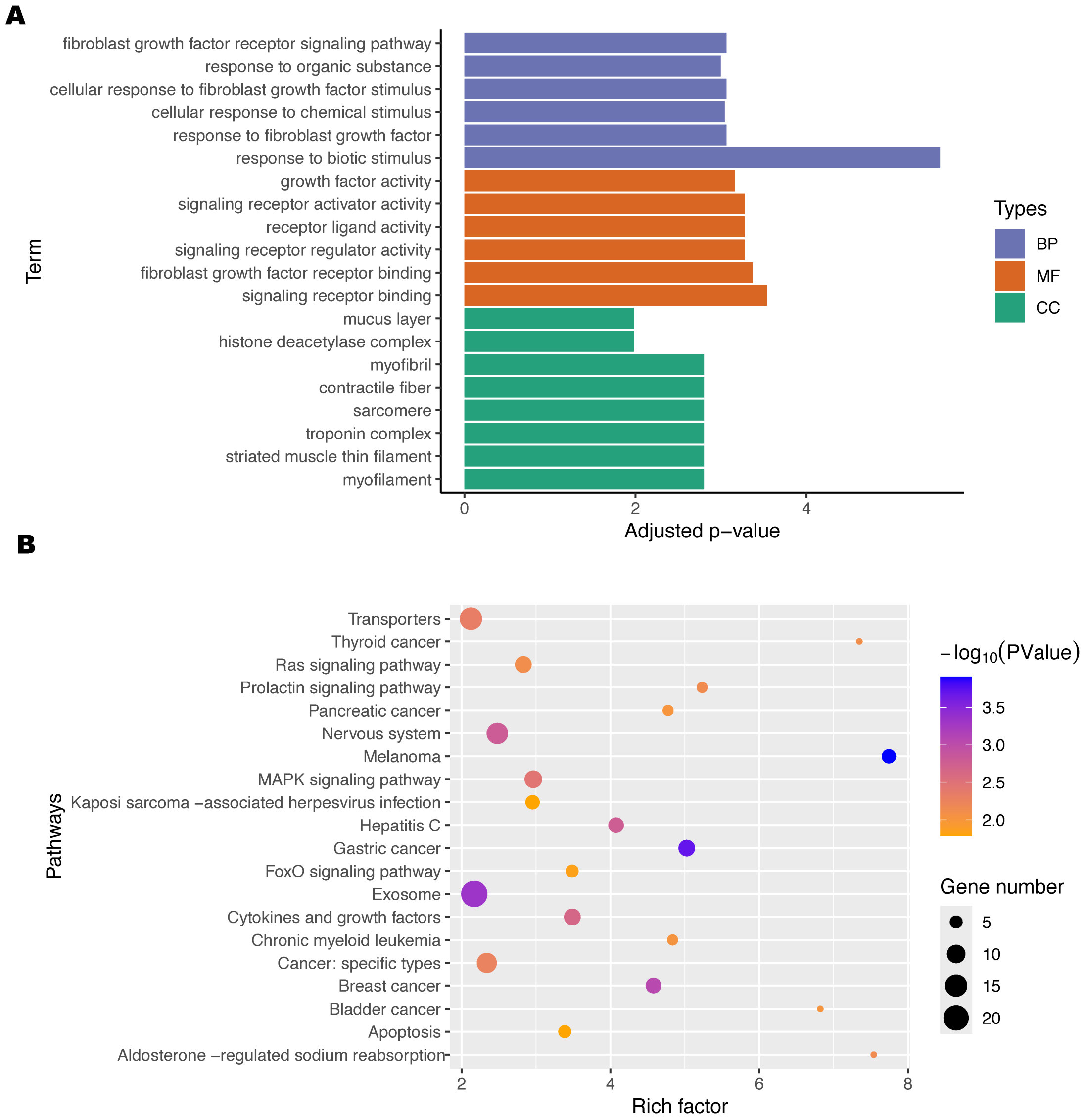

Supplementary Figure 2. GO terms bar plot and KEGG bubble plots. (A) Top 20 significant GO terms for backfat thickness. (B) Top 20 significant KEGG pathways for backfat thickness.

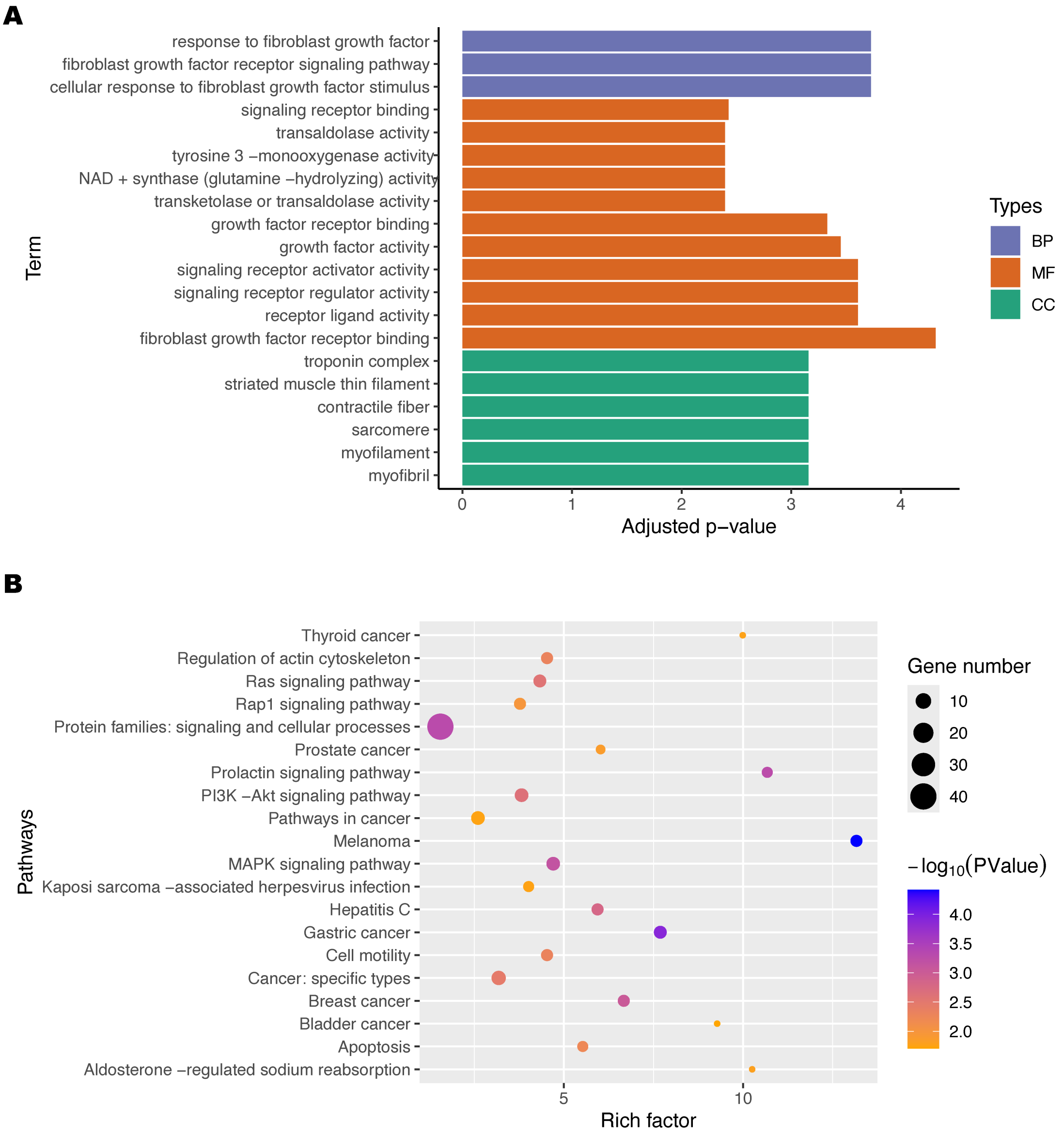

Supplementary Figure 3. GO terms bar plot and KEGG bubble plots. (A) Top 20 significant GO terms for feed conversion ratio. (B) Top 20 significant KEGG pathways for feed conversion ratio.

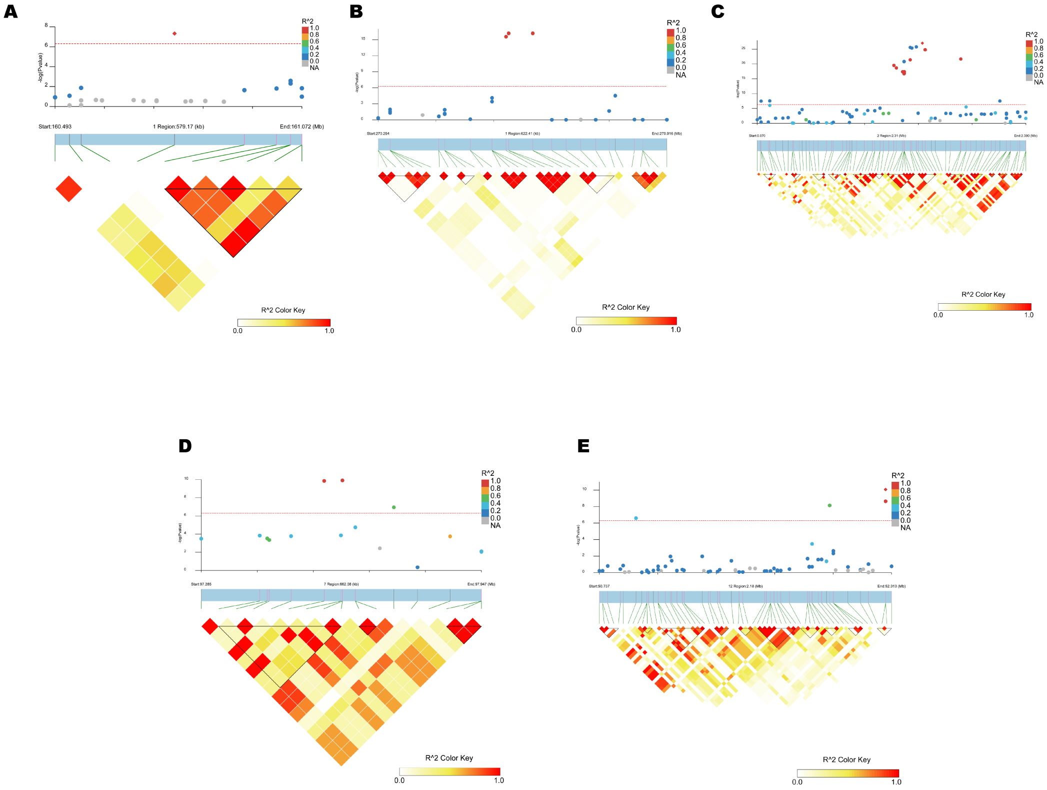

Supplementary Figure 4. Linkage disequilibrium plot for SNPs within the candidate regions for backfat thickness. (A) Linkage disequilibrium plot for SNPs within Chr1:160,473,437-161,073,437. The red line represents the Bonferroni cutoff which was 0.05/*N*, and *N* represents the number of variants used in the analysis. The legend on the right side represents the different R^2 of SNPs around the regions of peak SNP detected. (B) Linkage disequilibrium plot for SNPs within Chr1: 270,269,333-270,926,968. (C) Linkage disequilibrium plot for SNPs within Chr2: 0-2,457,364. (D) Linkage disequilibrium plot for SNPs within Chr7: 97,275,068-98,039,684. (E) Linkage disequilibrium plot for SNPs within Chr12: 50,709,260-53,168,040.

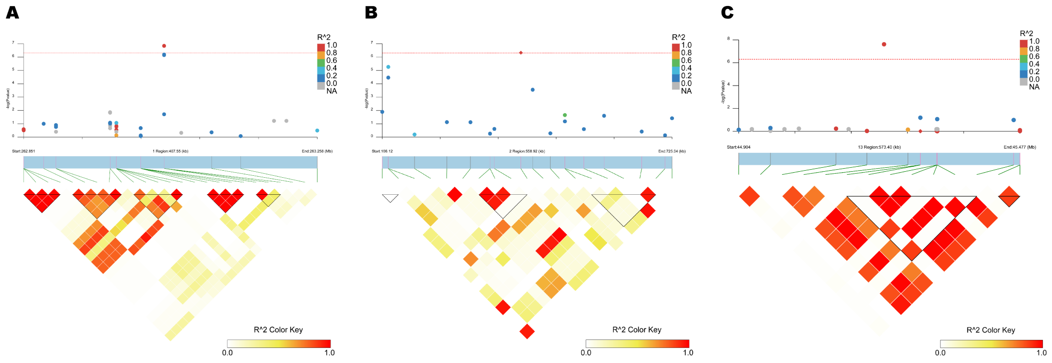

Supplementary Figure 5. Linkage disequilibrium plot for SNPs within the candidate regions for feed conversion ratio. (A) Linkage disequilibrium plot for SNPs within Chr1: 262,745,504-263,345,504. The red line represents the Bonferroni cutoff which was 0.05/*N*, and *N* represents the number of variants used in the analysis. The legend on the right side represents the different R^2 of SNPs around the regions of peak SNP detected. (B) Linkage disequilibrium plot for SNPs within Chr2: 133,461-733,461. (C) Linkage disequilibrium plot for SNPs within Chr13: 44,899,972-45,499,972.
